# A network analysis of sleep quality, cognitive function, and depressive symptoms among community-dwelling older adults

**DOI:** 10.3389/fpsyt.2026.1736786

**Published:** 2026-02-16

**Authors:** Youjie Yu, Han Cai

**Affiliations:** 1 Department of Psychiatry, School of Mental Health, Bengbu Medical University, Bengbu, China; 2 Department of Psychogeriatrics, The Fourth People's Hospital of Wuhu, Wuhu, China

**Keywords:** cognitive function, depression, network analysis, older adult, sleep quality

## Abstract

**Background:**

Many countries, including China, are facing rapid population aging issues. Among middle-aged and older adults, depressive symptoms and sleep disturbances are associated with cognitive impairment. Therefore, this study aimed to examine the relationships between sleep, depression, and cognitive function in older adults.

**Method:**

We recruited 457 elderly people aged over 60 years from the Matang community of Wuhu to participate in this cross-sectional study. We utilized the Mini-Mental State Examination (MMSE), Pittsburgh Sleep Quality Index (PSQI), and Geriatric Depression Scale (GDS) to measure our participants, with 126 individuals completing all of them. We used the collected data for network analysis to identify the bridge symptoms in the MMSE-PSQI-GDS network by calculating bridge centrality.

**Results:**

The core symptom of the MMSE, GDS, and PSQI networks was subjective sleep quality (SSQ). The bridge symptoms in MMSE, GDS, and PSQI were “GDS” and “OR” (orientation). Among the symptoms, “GDS” had the highest bridge centrality. The edge connecting nodes habitual sleep efficiency (HSE) and used sleep medication (USM) had the strongest weight (0.67) around all the symptoms of PSQI. Nodes attention and calculation (AC) and registration (RG) had the strongest weight (0.48), connecting all MMSE symptoms.

**Conclusions:**

This study highlights the central role of subjective sleep quality and the bridging role of depression in the interconnected network of cognition, sleep, and mood among older adults. These findings suggest that interventions targeting sleep quality and depressive symptoms may have broad benefits for cognitive function and mental health. By identifying key symptom interactions, this network-based approach provides guidance for prioritizing assessment and intervention strategies in the geriatric population.

## Introduction

Currently, the process of population aging is rapidly increasing worldwide, and the process of population aging in China is faster than that in other medium- and low-income countries (1). According to this prediction, China has been considered an aged society since 2022 and will become a super-aged society by 2033 (2). Based on the seventh national census data in 2021, the population over 60 years old was approximately 260 million, consisting of 18.7% of the total population, an increase of 5.44 percentage points compared with that in 2010 (1).

With the increasing aging population, the issue of maintaining the health of the elderly has become more pressing. Cognitive function has been identified as one of the most significant factors in healthy aging (3). Therefore, it is essential to identify risk factors related to cognitive function to prevent and delay dementia (4). In addition, the prevalence of mild cognitive impairment in individuals aged 65 years and older is currently 20.8% in China (5). The prevalence rate of sleep disturbance in older adults is 10.4%–62.1% (6). According to some studies, cognitive function impairment in adults is related to poor sleep quality because sleep can influence various psychological and neural functions, including cognitive function (7). Furthermore, the relationship between sleep quality and psychological health has been confirmed (4). Systematic reviews have shown that the relationship between sleep quality and depression is bidirectional; thus, sleep quality predicts depression and vice versa (8). Depression is the most common emotional disturbance (9). Due to the influence of many factors, such as physical aging, chronic diseases, declining social relationships, and economic status, depression has become a common psychological disorder among older adults (10–12). Research shows that the overall prevalence of depression was 10.2% among older Chinese adults aged 65 and older (13). Moreover, depression may play a connecting role between sleep quality and cognitive function in older adults (4). Therefore, aging increases the risk of depression, which can, in turn, cause sleep disturbances and amplify their negative effects on cognition. Sleep disruptions, such as fragmented sleep, which may trigger systemic inflammation, contribute to cognitive decline.

The network theory of psychopathology offers a conceptual framework that conceptualizes mental disorders as systems of interacting symptoms rather than manifestations of a single latent disease entity. Within this framework, symptoms are assumed to mutually influence and reinforce one another, and certain symptoms may play a central role in maintaining the entire system or serve as bridges linking different domains. This approach is particularly relevant in older adults, in whom sleep disturbances, cognitive decline, and depression symptoms frequently co-occur and interact in complex and dynamic ways. However, to date, no study has integrated sleep, cognition, and depressive symptoms into a symptom-level network analysis. Compared with variable-level analytical approaches that examine unidirectional associations between total scale scores, such as regression or mediation models, network analysis can identify core and bridge symptoms, thereby providing clinically meaningful insights for prioritizing assessment and intervention targets. In addition, cognitive function can be assessed using interview- or performance-based measures. The former captures subjective experience but is less standardized, while the latter provides objective, standardized evaluation but is influenced by task complexity and administration. In this study, we integrated both approaches to examine the interplay between cognition, depression, and sleep in older adults.

Therefore, the objectives of the present study were as follows: (1) to construct a symptom-level network integrating cognitive function, sleep quality, and depressive symptoms among community-dwelling older adults and (2) to identify core and bridge symptoms that may link cognitive impairment, sleep disturbances, and depressive symptoms within this network.

## Methods

### Participants

A total of 457 older adults entered the study, of whom 126 completed the questionnaires. The completed questionnaires were included in the analysis; the mean participant age was 72.33 years (SD = 4.99). Among them, 62 (49.2%) were male and 64 (50.8%) were female. The data are shown in **Table 1**.

**Table 1:** Distribution of recruited participants by gender, cognitive impairment, depression severity, and sleep status.

Demographic characteristics (N = 126)			
Variables		N	%
Gender	female	64	50.8
	male	62	49.2
MMSE	cognitive impairment	89	70.6
	without cognitive impairment	37	29.4
GDS	mild depression	24	19.0
	severe depression	4	3.2
	normal	98	77.8
PSQI	normal	32	25.4
	sleep disorder	94	74.6

### Measures

#### Mini-mental state examination

According to the definition in the Diagnostic and Statistical Manual of Mental Disorders (DSM), MMSE is commonly used as part of the process to evaluate a potential diagnosis of dementia. The MMSE is a paper-based test with a maximum score of 30. It includes a test regarding time and place, repeating lists of words, arithmetic problems such as repeated subtractions, language use and comprehension, and basic motor skills. Lower scores indicate more severe cognitive problems (14).

#### Pittsburgh sleep quality index

The PSQI is a self-rated questionnaire that comprises both fixed-choice and open-ended questions. It can evaluate several aspects of sleep, such as sleep quality and disturbances, over a 1-month time interval. It yields scores for seven distinct components: (1) subjective sleep quality, (2) sleep latency, (3) sleep duration, (4) habitual sleep efficiency, (5) sleep disturbances, (6) use of sleeping medication, and (7) daytime dysfunction (15).

#### Geriatric Depression Scale

The Geriatric Depression Scale (GDS) was developed in 1982 as a self-report screening tool to detect depression among older adults. First, researchers selected 100 questions believed to have the potential to distinguish elderly individuals with depression from healthy subjects, with various elements addressing cognitive complaints, motivation, future/past orientation, personal mood, etc. Thirty questions with the highest correlations with the total score were chosen to create the GDS-30 scale (16).

### Procedure

Participants were recruited through convenience sampling during health check-ups in the Matang community from January to August 2023. All participants were informed that their responses would be anonymous, confidential, and used solely for academic research purposes. The inclusion criteria were as follows: (1) age ≥65 years and (2) ability to complete a face-to-face interview. The exclusion criteria were as follows: (1) inability to complete key items of the MMSE, PSQI, or GDS; and (2) missing data that were unsuitable for network analysis. A total of 457 individuals were initially recruited, of whom 126 participants who completed all questionnaires were included in the final analysis, while 331 participants were excluded due to incomplete data.

### Network construction

Network analysis was conducted using R (version 4.3.3). A Gaussian graphical model (GGM) was estimated based on automatically selected correlations using the *cor_auto* function, which is suitable for mixed continuous and ordinal data. Nodes represented MMSE cognitive subdomains, PSQI sleep components, and the total GDS score, entered using the original scoring metrics without standardization to preserve clinical interpretability. The network was estimated using regularized partial correlations and visualized with the default spring layout implemented in the *qgraph* package based on the Fruchterman–Reingold algorithm. Edges represent conditional associations between nodes, with edge thickness indicating the association strength and color indicating the direction. To ensure data integrity, missing values were handled using listwise deletion.

### Network accuracy and stability estimates

The accuracy of the edge and stability estimates for the network was calculated using a bootstrapping procedure with 1,000 iterations. First, we estimated the accuracy of the edge using the 95% confidence interval (CI) of the bootstrapped edge weight. A lower overlap between the CIs indicates higher accuracy. Second, we tested the stability of node centrality using subset bootstrapping. We estimated the centrality stability coefficient (CS coefficient) as a reference. For the CS coefficient, values greater than or equal to 0.25 indicated acceptable stability, and values greater than or equal to 0.5 indicated good stability.

## Results

### Constructed symptom network of the MMSE, PSQI, and GDS

**Figure 1:**
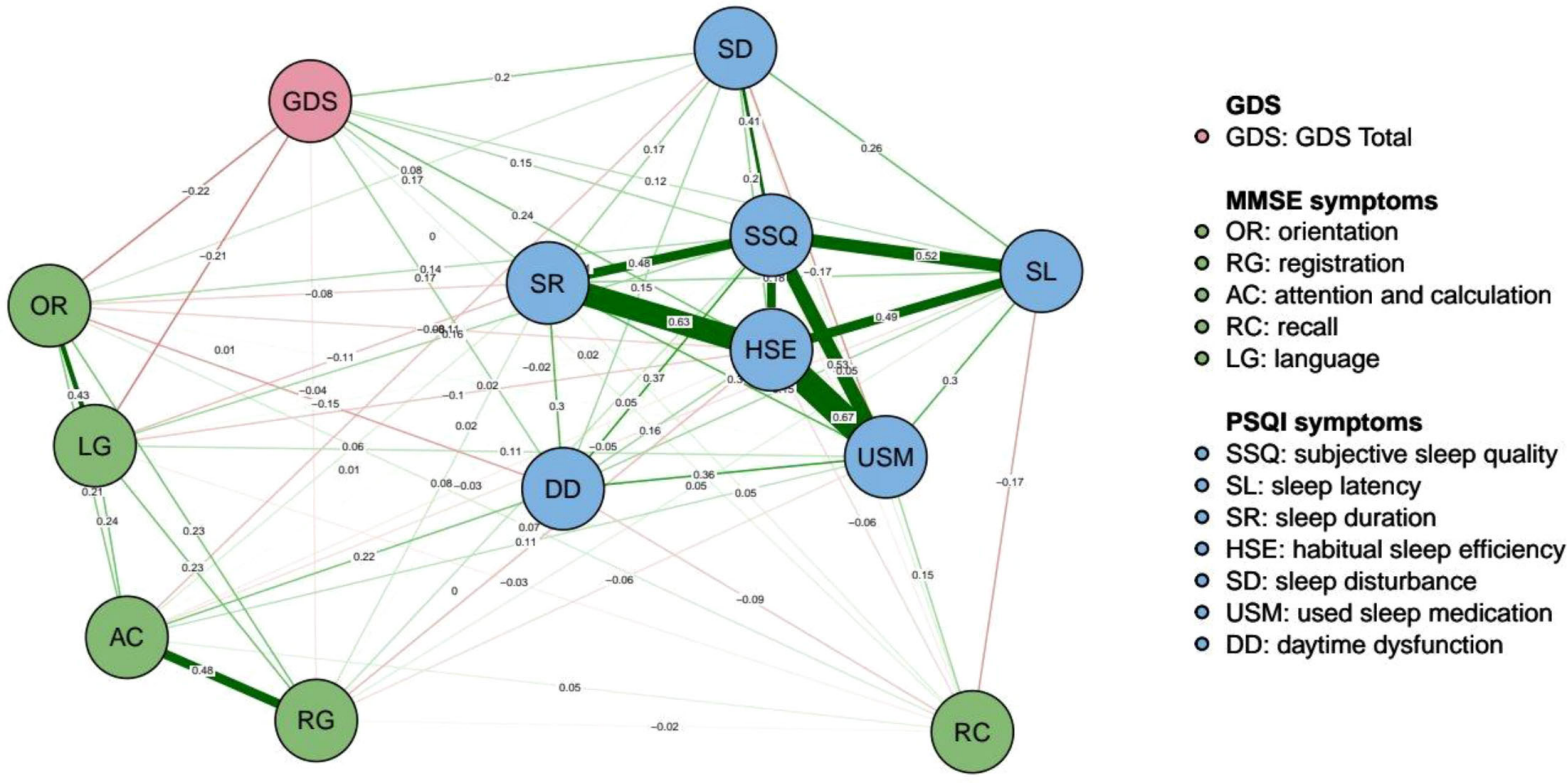
Network structure of sleep quality, cognitive function, and depressive symptoms among ommunity-dwelling older adults. Positive correlations are shown in green, and negative correlations in red. Thicker and more intense edges indicate stronger correlations.

**Figure 2:**
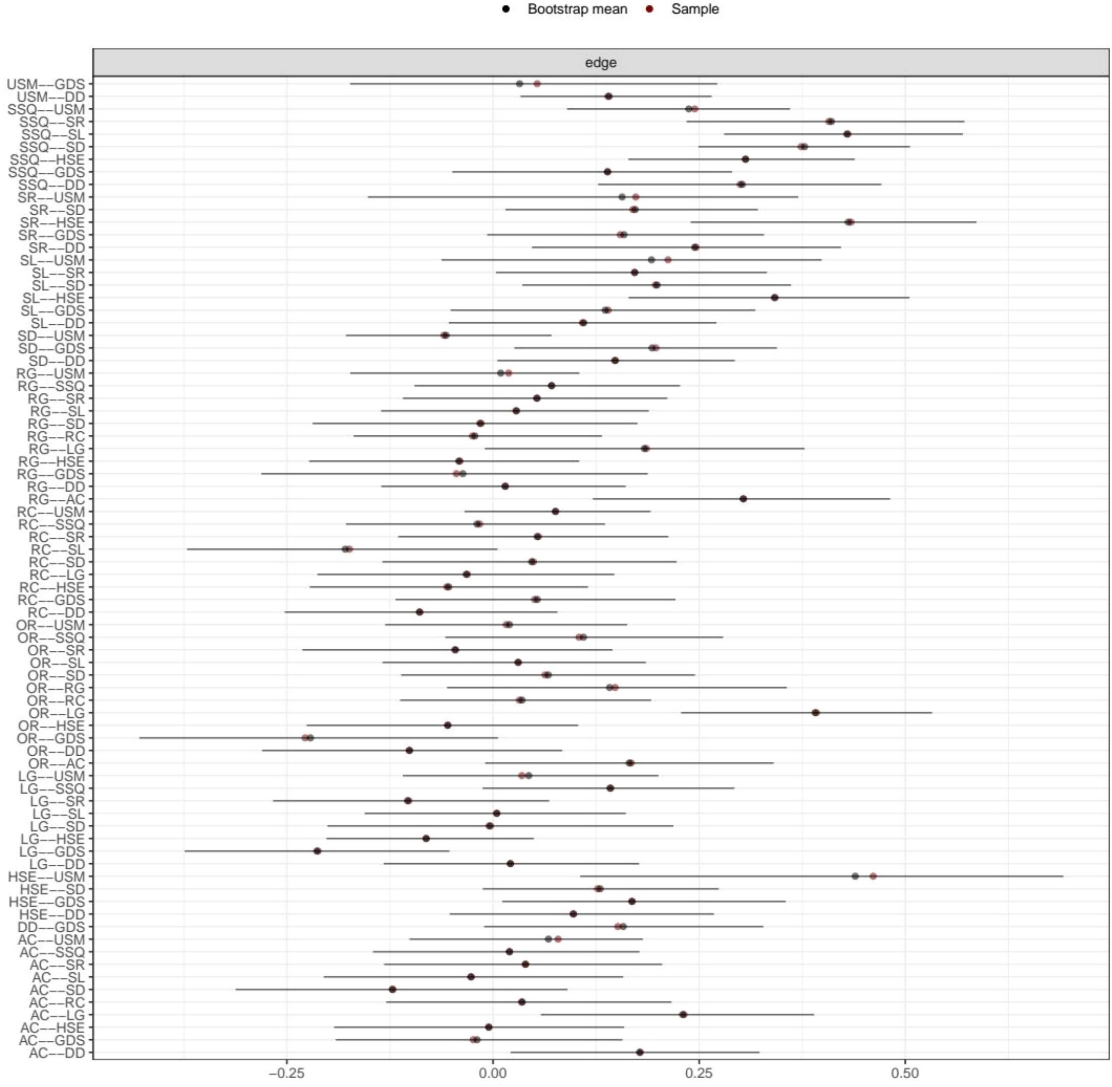
Nonparametric bootstrap confidence intervals for edge weights in the network. Red points indicate edge weight estimates from the original sample, and black points represent the bootstrap means.

The estimated network, including 13 nodes, five components of the MMSE, seven items of the PSQI, and one total score of the GDS, is shown in **Figure 1**. The estimated network yielded 78 edges (13 * (13 − 1)/2), all of which had non-zero weights (mean weight 0.124). The weight of the edge connecting “HSE” and “USM” was the strongest (0.67). Other strong associations included those between “SR” and “HSE” (0.63) and between “SSQ” and “USM” (0.53). The edge connecting nodes “HSE” and “USM” also had the strongest weight (0.67) for all the PSQI symptoms. Nodes “AC” and “RG” had the strongest weight (0.48), connecting all the MMSE symptoms. **Figure 2** shows the 95% confidence intervals for the edge weights.

### Network centrality of the MMSE–PSQI–GDS network

**Figure 3:**
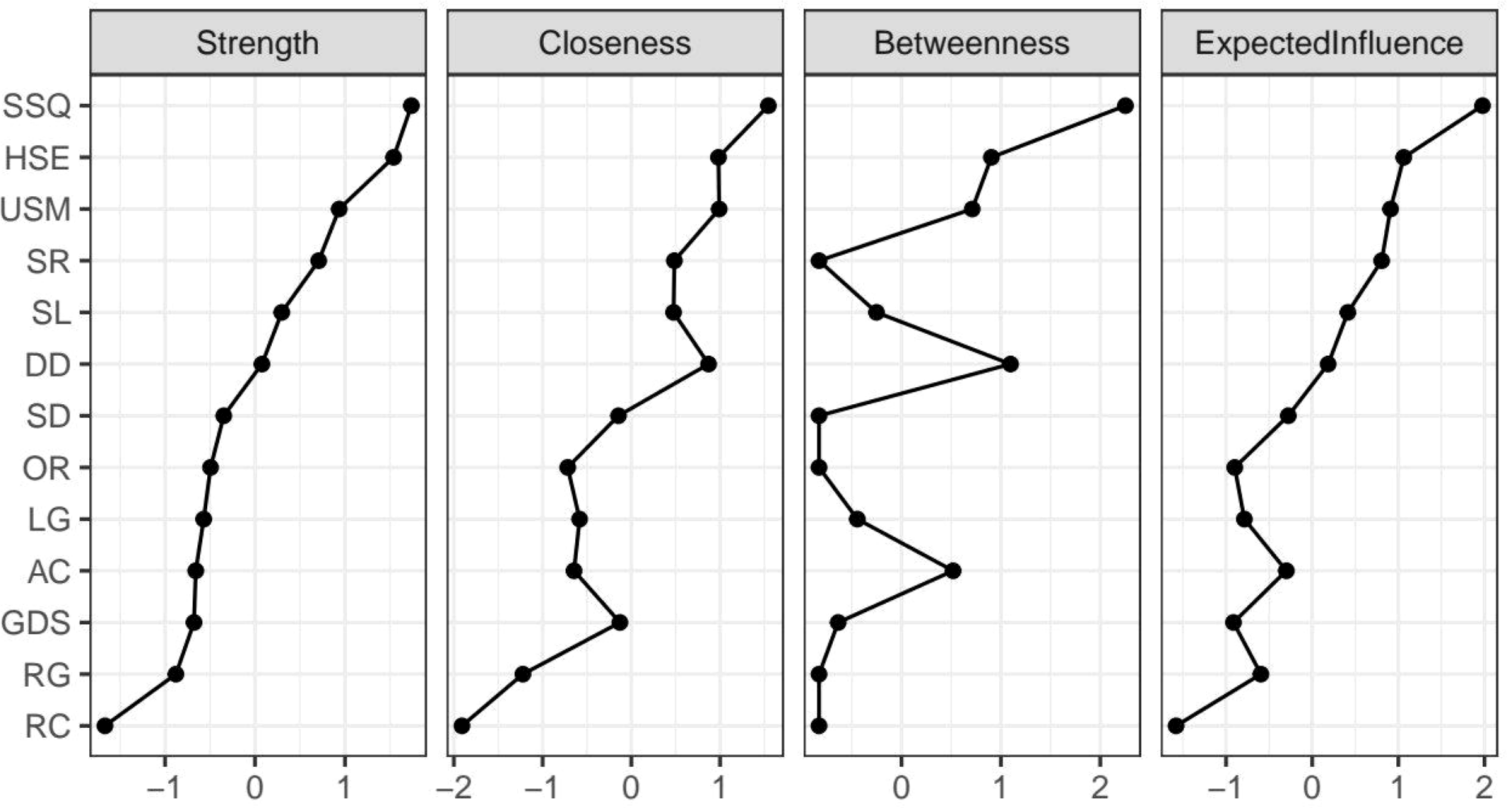
Centrality measures of each symptom in the network, including strength, closeness, betweenness, and expected influence.

**Figure 4:**
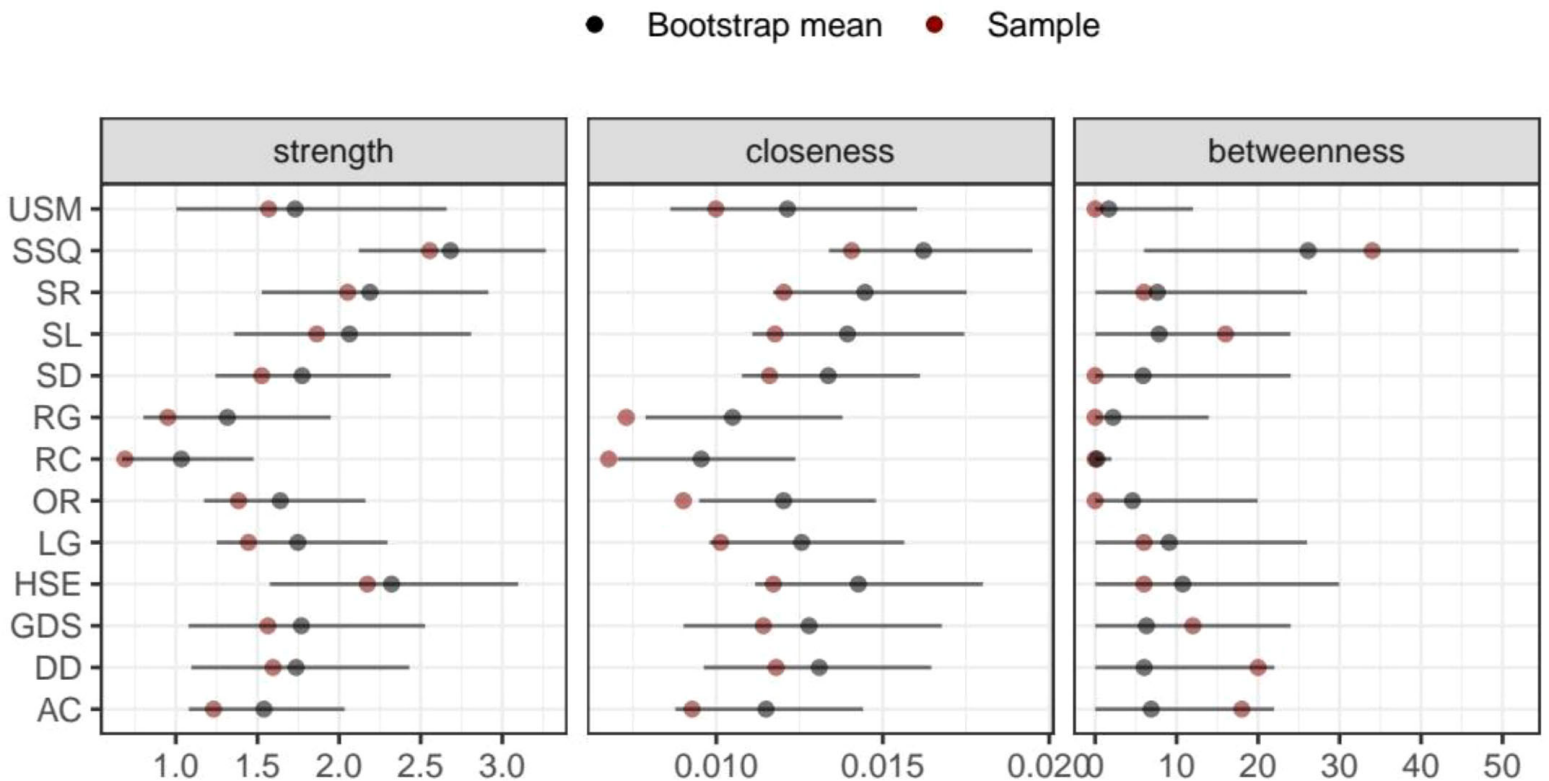
Nonparametric bootstrap confidence intervals for node centrality estimates in the network. Red points indicate estimates from the original sample, and black points represent the bootstrap means.

The network centrality indices of the 13 symptoms are shown in **Figure 3**. Node “SSQ” was the core symptom, displaying the highest strength, betweenness, and Expected Influence (EI). The highest strength proved that node “SSQ” had the most potent effect on the other nodes, regardless of direction. The highest betweenness indicated that “SSQ” appeared most frequently on the paths between different subsystems, serving as a crucial conduit for information transmission and activation spread. The highest EI of node “SSQ” indicated that this node was the strongest predictor in the network and was linked to the entire symptom network. Strength, betweenness, and EI reflected a node’s total level of involvement in the network. The highest strength, betweenness, and EI of node “SSQ” further proved its core role in the network. **Figure 4** shows the 95% confidence intervals for node centralities.

### Bridge centrality of the MMSE–PSQI–GDS network

**Figure 5:**
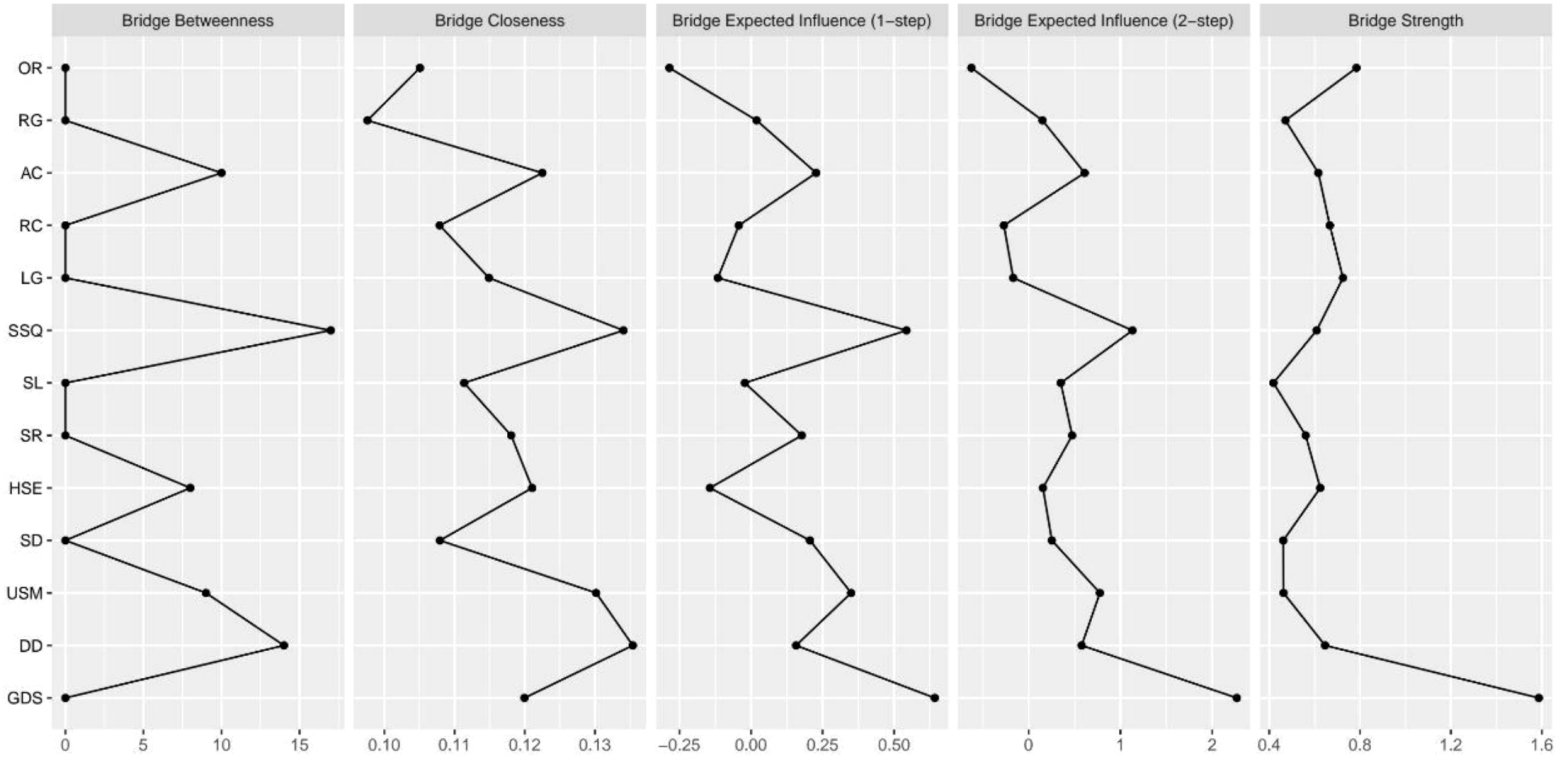
Standard bridge centrality measures of sleep quality, cognitive function, and depressive symptoms among community-dwelling older adults.

**Figure 5** shows two nodes (“GDS,” “OR”) with bridge strengths in the top 10%. These nodes were classified into the bridge group. Node “GDS” exhibited the highest network bridge expected influence and bridge strength, while it was closer to the average in its strength and expected influence of network centrality. However, “SSQ” exhibited the highest network bridge betweenness, and it was also the highest in terms of betweenness of network centrality. The edge connecting “USM” had the strongest weight (0.53) with node “SSQ” in the PSQI community, while the edge connecting “LG” had the strongest weight (0.16) in the MMSE community. In addition, the edge connecting “HSE” had the strongest weight (0.24) with node “GDS” in the PSQI community, while the edge connecting “OR” had the strongest weight (−0.22) in the MMSE community. Although “OR” had the second highest network bridge strength, it did not play a prominent role in other aspects.

### Accuracy and stability of the MMSE–PSQI–GDS network

**Figure 6:**
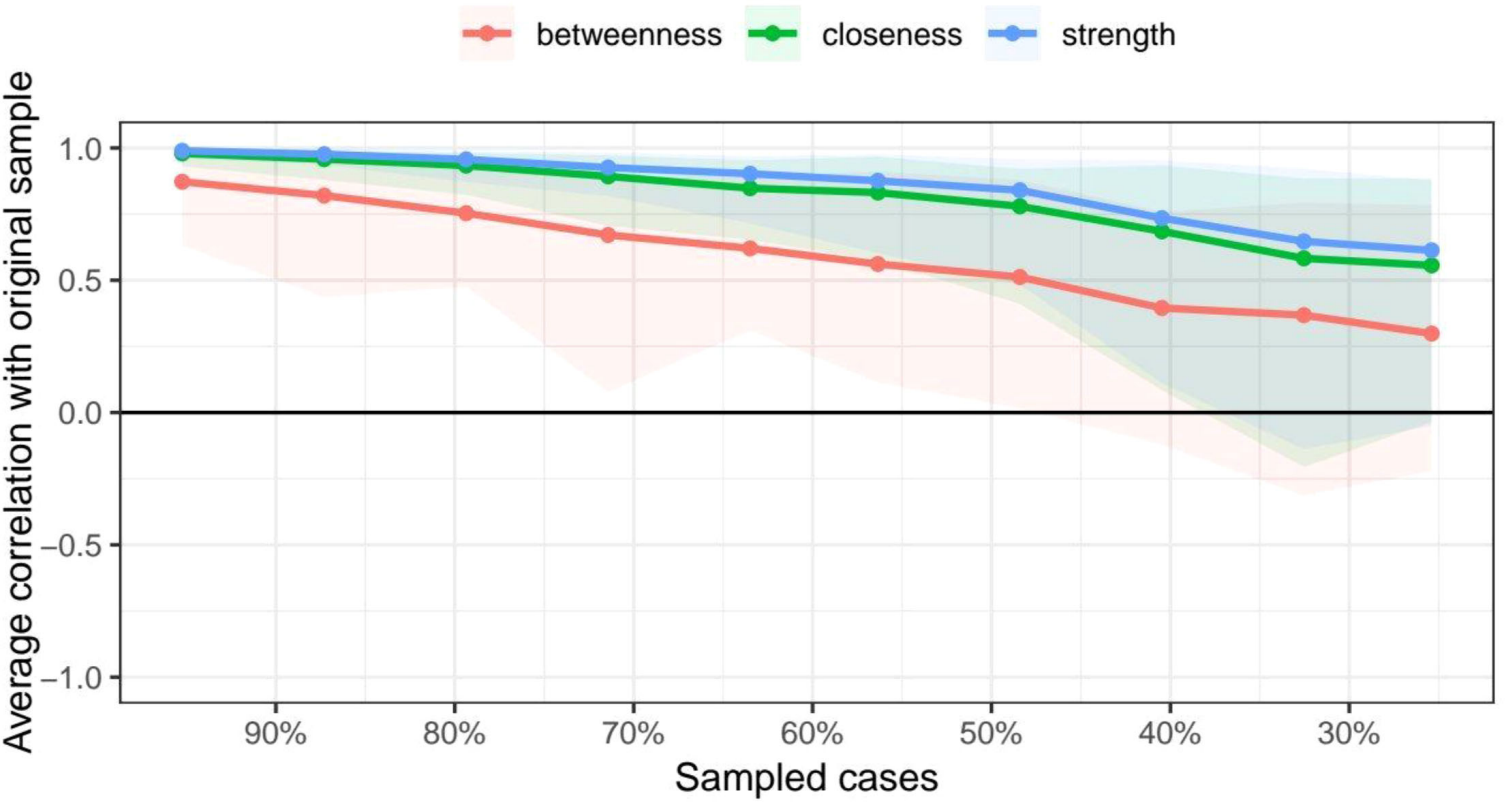
Stability of centrality indices, including betweenness, closeness, and strength. The red, green, and blue lines represent the average correlations between the sampled networks (with cases excluded) and the original network for betweenness, closeness, and strength, respectively.

The average edge weights evaluated were consistent with the edge weights of the samples in this study. This showed that the edge weight had sufficient stability via edge weight bootstrapping. **Figure 6** shows the results for the CS coefficient index. The CS coefficient index of closeness was 0.365, the CS coefficient index of edge was 0.516, and the CS coefficient index of strength was 0.437. This result from the stability analyses indicated that the network models were relatively stable.

## Discussion

This study used network analysis to indicate the network structure of the symptoms of older adults’ cognition, depression and sleep disturbance to identify the interactions between symptoms. Core and bridge symptoms were identified in the MMSE–PSQI–GDS network.

The results showed that the core symptom in the network was subjective sleep quality (SSQ), indicating that it was most strongly associated with other symptoms and occupied a central position in the network structure. “SSQ” exhibited the highest node strength, betweenness centrality, and bridge betweenness, suggesting close associations with cognitive function, depressive symptoms, and sleep-related variables. Its highest expected influence further indicated that subjective sleep quality was involved in the strongest overall pattern of co-activation or co-inhibition with other variables in the network, and variations in the SSQ were associated with broader system-level variations. This finding is consistent with previous evidence identifying subjective sleep quality as a central complaint in major depressive disorder (17). We hypothesized that insufficient sleep may be associated with increased levels of Aβ in the brain, thereby promoting amyloid plaque formation and potentially affecting cognitive function (18). Although prior studies have reported no significant associations between subjective or objective sleep measures and global cognition assessed using the MMSE (19), this does not preclude potential interrelations at the symptom level. Other studies have suggested that poor sleep quality is associated with greater neurocognitive impairment in patients with major depressive disorders (20). Therefore, low-to-moderate intensity physical activity interventions, such as walking or Tai Chi, may be considered feasible approaches to improve subjective sleep quality in older adults (21).

Notably, although the “GDS” exhibited only moderate levels of node strength and expected influence within the overall network, it stood out in bridge centrality measures, ranking highest in both bridge strength and bridge expected influence. This finding suggests that depressive symptoms may be closely connected with both sleep- and cognition-related domains and may occupy a key position linking symptom clusters across systems. Previous studies have also demonstrated that sleep efficiency is closely associated with the risk of depression (22). Consistent with these findings, the present study observed a positive association between habitual sleep efficiency and depression symptoms. In addition, increasing depressive symptoms have been shown to be associated with a more rapid decline in cognitive function among Chinese older adults, particularly in orientation and language domains (23). Moreover, loneliness and social isolation have been reported to increase the risk of depression in older populations (24). Therefore, regular community-based activities that enhance social participation may help reduce depressive symptoms and potentially contribute to concurrent improvements in sleep and cognitive functioning.

Based on the edge weight analysis, the strongest association was observed between habitual sleep efficiency (HSE) and use of sleep medication (USM), with a weight of 0.67. This finding suggests that lower sleep efficiency tends to co-occur with greater use of sleep medication, indicating a potentially reinforcing association between poor sleep patterns and pharmacological coping strategies. Another relatively strong connection was identified between sleep duration (SD) and HSE, indicating that habitual sleep efficiency is closely correlated with sleep duration. This pattern implies that sleep-related symptoms are highly interconnected and that focusing on a single symptom in isolation may be insufficient, highlighting the relevance of considering overall sleep behavior patterns. In addition, within the MMSE symptom network, the strongest edge weight was observed between attention and calculation (AC) and registration (RG), suggesting a close association between basic attentional functioning and immediate memory processing. This association may reflect the interdependence of attentional resources and early stage memory processes, as attention is closely related to information encoding, and registration represents the initial stage of short-term information retention. Accordingly, lower performance in attention-related domains tends to be accompanied by poorer immediate memory performance, which may be linked to broader patterns of cognitive function.

The CS coefficient index of betweenness in this network was low, indicating that the stability of betweenness was not high. Therefore, the conclusions drawn from the betweenness analysis may be biased. However, aside from having the highest betweenness, SSQ was also the core node of the entire network. This suggests that its role in the network remains significant. In addition, the CS coefficient index of strength, closeness, and expected influence all exceed 0.25, which qualifies as an acceptable stability for these centrality metrics. Nonetheless, the overall CS coefficients are relatively low, which might be due to the limited sample size. Future research should expand the sample size to improve the stability and reliability of the findings.

### Limitation

First, the cross-sectional design of this study precludes any causal inference, and longitudinal studies are required to clarify the potential causal relationships. Second, participants were recruited using convenience sampling, which may limit the generalizability of the findings and reduce sample representativeness. In addition, because the assessment battery was relatively time-consuming for older adults, many participants were unable to complete all the questionnaires, resulting in substantial data attrition. Consequently, the final sample with complete data was relatively small for the requirements of network analysis, which may have affected the stability and accuracy of the estimated networks. Future studies should consider more efficient and feasible assessment strategies to improve the completion rates in older populations. Moreover, the variables were entered into the network using their original scoring metrics and were not standardized to preserve clinical interpretability. While this approach enhances clinical relevance, differences in scale ranges may have influenced the edge weights and centrality estimates, potentially affecting comparisons across nodes. Future studies may benefit from sensitivity analyses comparing standardized and nonstandardized networks. Finally, due to the limited sample size, depressive symptoms were included as the total GDS score rather than individual items to meet the methodological requirements. This may have obscured the symptom-level heterogeneity and introduced bias into the network structure. Larger samples are needed in future research to examine the interrelations among individual depressive symptoms in greater detail.

## Data Availability

The raw data supporting the conclusions of this article will be made available by the authors, without undue reservation.
